# Isolation of Two Novel Human Anti-CTLA-4 mAbs with Intriguing Biological Properties on Tumor and NK Cells

**DOI:** 10.3390/cancers12082204

**Published:** 2020-08-06

**Authors:** Margherita Passariello, Cinzia Vetrei, Emanuele Sasso, Guendalina Froechlich, Chiara Gentile, Anna Morena D’Alise, Nicola Zambrano, Elisa Scarselli, Alfredo Nicosia, Claudia De Lorenzo

**Affiliations:** 1 Department of Molecular Medicine and Medical Biotechnology, University of Naples “Federico II”, Via Pansini 5, 80131 Napoli, Italy; margherita.passariello@unina.it (M.P.); cinzia.vetrei@unina.it (C.V.); chi.gentile27@gmail.com (C.G.); zambrano@unina.it (N.Z.); nicosia@ceinge.unina.it (A.N.); 2 Ceinge—Biotecnologie Avanzate S.C. A.R.L., via Gaetano Salvatore 486, 80145 Naples, Italy; guendalinafrex@gmail.com; 3 Nouscom srl, Via di Castel Romano 100, 00128 Rome, Italy; emanuelesasso90@gmail.com (E.S.); m.dalise@nouscom.com (A.M.D.); E.Scarselli@nouscom.com (E.S.); 4 European School of Molecular Medicine, University of Milan, Via Festa del Perdono 7, 20122 Milan, Italy

**Keywords:** CTLA-4, immune checkpoint, immunomodulatory mAbs, cancer immunotherapy, natural killer cells

## Abstract

The cytotoxic T lymphocyte-antigen 4 (CTLA-4) has been considered an IC exclusively expressed on T cells, where it counteracts the co-stimulatory CD28 receptor, by competing for its binding to CD-80 and CD-86. We recently found that it is expressed also on tumor and NK cells, suggesting other possible unknown roles of CTLA-4. To shed light on these novel aspects of CTLA-4, we used Ipilimumab, the first FDA approved human antibody targeting CTLA-4, in parallel studies with two novel human mAbs we isolated by using an efficient phage display selection strategy on live activated lymphocytes and purified mouse and human CTLA-4. The selection for cross-reactive mAbs was guaranteed by a high throughput sequencing to identify the sequences commonly enriched by two parallel pannings on human and mouse CTLA-4. Two isolated antibodies were found to bind with high affinity to both human and mouse CTLA-4 and lymphocytes, showing nanomolar or sub-nanomolar Kd values. They were able to kill Treg cells by ADCC, and to activate both human and mouse PBMCs, by strongly increasing cytokines secretion. Interestingly, they activated NK cells, exhibited cytotoxicity against cancer cells by inducing ADCC and inhibited tumor cell growth by affecting CTLA-4 downstream pathways in a similar fashion to CD-80 and CD-86 ligands and differently from Ipilimumab. Moreover, the novel mAbs showed a reduced ability to interfere in the binding of CD-80 ligands to CTLA-4 on T cells with respect to Ipilimumab, suggesting that they could allow for anti-tumor effects without the irAEs associated with the potent antagonistic activity of Ipilimumab.

## 1. Introduction

Cancer immunotherapy includes multiple approaches aimed at improving immune responses against cancer cells. Among them, the antibody-based approach specifically targets either antigens overexpressed on cancer cell surface for inhibiting their growth or immunecheckpoints (ICs), key regulators of immune cells signaling pathways responsible for self-tolerance, and for activating T cells against cancer cells [1,2,3].

The cytotoxic T lymphocyte-antigen 4 (CTLA-4), also known as CD152, is a very interesting IC, as it plays a crucial role in T cell inhibition [4]. Indeed, it is well known that inactivation of CTLA-4 in mice and in humans [5,6] induces hyperactivation of lymphocytes leading to autoimmune diseases due to the loss of its negative regulation. It has been proposed that the negative signal delivered by CTLA-4 on T cells, where it is mainly present as an intracellular protein, but transiently exposed on the surface of activated T cells [7], is due to its high affinity binding for B7-1/B7-2 ligands [8] on antigen-presenting cells [9,10], thus interfering in their interaction with the T cell-associated CD28 receptor. Since the co-stimulatory CD28 signal is necessary for full T cell activation and survival, CTLA-4 engagement mediates a negative regulation of immune response, by inhibiting T cell receptor (TCR) associated pathways [11].

Moreover, being constitutively expressed on regulatory T cells (Treg), CTLA-4 acts differently on this cell population to impair immune response: It reduces, on Treg, the stimulatory function [12,13] of B7-1 and B7-2, by mediating their removal through trans-endocytosis. In addition to Effector T cells and T reg cells, that have been considered for a long time the only populations expressing CTLA-4, it has been recently reported by our group that also natural killer (NK) cells display CTLA-4 on their surface, even though its role has not been yet fully elucidated [5,6]. Furthermore, as reported in recent studies, other types of cells, such as leukemia and solid tumor cells [14,15,16,17], express this protein on their membrane. Despite the precise function of CTLA-4 expressed on non-immune cells is largely unknown, some conflicting evidences indicate that it can affect tumor cell survival, by sustaining the proliferation or inducing apoptosis [15,16,18]. Indeed, CTLA-4 downstream signaling pathway has not been yet clarified, but it is well known that the cytoplasmatic tail of this protein contains two tyrosine residues that can act as binding sites for phosphatases or other intracellular adaptor proteins [19]. When unphosphorylated this tyr motif can be recognized by Clathrin adaptor complexes AP1 and AP2, regulating the intracellular concentration of CTLA-4 [20] and its internalization from the plasma membrane [21,22,23]. On the contrary, CTLA-4 phosphorylation, mediated in T cells by src kinases p56lck and p59fyn [21,24,25] not only is essential to avoid internalization [26], but it also creates harbors for SH2 domain of phosphatases [27] and for other intracellular proteins such as the p85 subunit of phosphatidylinositol 3 kinase (PI3K) [19,26]. Since the AP-2 binding site in CTLA-4 overlaps with that of PI 3-kinase and SHP-2, it is likely that the tyrosine phosphorylation would facilitate the binding of PI3-kinase and SHP-2 by displacing the AP-2 association which is independent of tyrosine phosphorylation.

The discovery that CTLA-4 inhibits T cell activation made it an attractive target for cancer immunotherapy, leading to the development of inhibitory antibodies blocking its function. Ipilimumab was the first fully human anti-CTLA-4 mAb proposed by Allison’s research group in 1996 [28] and approved by the U.S. Food and Drug Administration (FDA) in 2011 for the treatment of metastatic melanoma [29,30]. Furthermore, several clinical trials have been started for other solid tumors, such as non-small-cell lung cancer (NSCLC), and renal cell and prostate carcinomas [31], by testing Ipilimumab alone or in combination with other treatments leading to its approval in 2019 by European Medicines Agency for the treatment of renal cell carcinoma and by FDA for the treatment of metastatic colorectal cancer in combination with Nivolumab [32,33]. Recently, it has been proposed that the therapeutic effect of Ipilimumab may be based not only on its ability to block the interaction of CD28 with CD-80 and CD-86 but also on its effects on Tregs by inhibiting the function of CTLA-4 on Tregs and by inducing their efficient elimination in the tumors by Antibody Dependent Cell-Mediated Cytotoxicity (ADCC) [34,35].

Unfortunately, despite its great therapeutic success mainly for metastatic melanoma, 60% of patients treated with Ipilimumab showed immune related adverse events (irAEs), with severe toxicities in about 10–15% of cases [36,37].

Herein we describe the isolation of two novel human mAbs, selected by phage display [38] with an innovative strategy aimed at cross-reactivity for human-mouse CTLA-4 in order to allow for studies in different models useful for a better elucidation of the new roles played by CTLA-4 on the different immune and non-immune cell populations. We found that these novel antibodies were as effective as Ipilimumab in activating PBMCs and eliminating Treg by ADCC, but they also showed the ability to activate NK cells more efficiently than Ipilimumab and to inhibit tumor cell growth with a different mechanism of action from that of Ipilimumab. In particular, we show here for the first time that they activate different pathways downstream CTLA-4 in tumor and NK cells, shedding light also on unrevealed potential roles of this receptor on different cell populations. Furthermore, differently from Ipilimumab, the novel isolated mAbs have a reduced ability to interfere in the binding of ligands to CTLA-4 on T cells, thus we argue that their therapeutic effect could be distinct from the antagonistic activity of Ipilimumab which is also considered responsible for irAEs [39].

## 2. Results

### 2.1. Selection of Human/Mouse CTLA-4 Crossreactive Antibodies

The strategy used for the isolation of anti-CTLA-4 scFv-phages consisted in alternate panning rounds on live activated hPBMCs expressing the target protein and on recombinant purified human or mouse chimeric CTLA-4-Fc. This approach was devised to guarantee an efficient selection of a large number of clones with a high specificity for CTLA-4 in its native conformation, as that presented on the cell membrane, and endowed with cross reactivity for the mouse protein in order to allow for their use in both the species.

Phage particles from a Phagemid library of up to 10^10^ different clones were prepared as described in Materials and Methods. In the first selection round, human PBMCs activated with Dynabeads Human T-Activator CD3/CD28 for 96 h, as described in Materials and Methods, were used as antigen-positive cells.

In the second and third panning rounds, recombinant human or mouse chimeric CTLA-4-Fc protein, coated on tubes, were used in parallel as the bait for the selections. These two latter selection rounds consisted in two successive negative pannings on the Fc, followed by a positive selection on each chimeric fusion protein-Fc, to subtract the phages recognizing the Fc domain present in the chimeric proteins.

In each round of selection after 16 h of incubation at 4 °C, the tubes either containing CTLA-4 positive cells or coated with the recombinant chimeric protein were extensively washed and phages bound to the cell surface or to the coated protein were eluted by using a low pH buffer, and used to infect *Escherichia coli* TG1 for amplification and further selection rounds.

**Figure 1:**
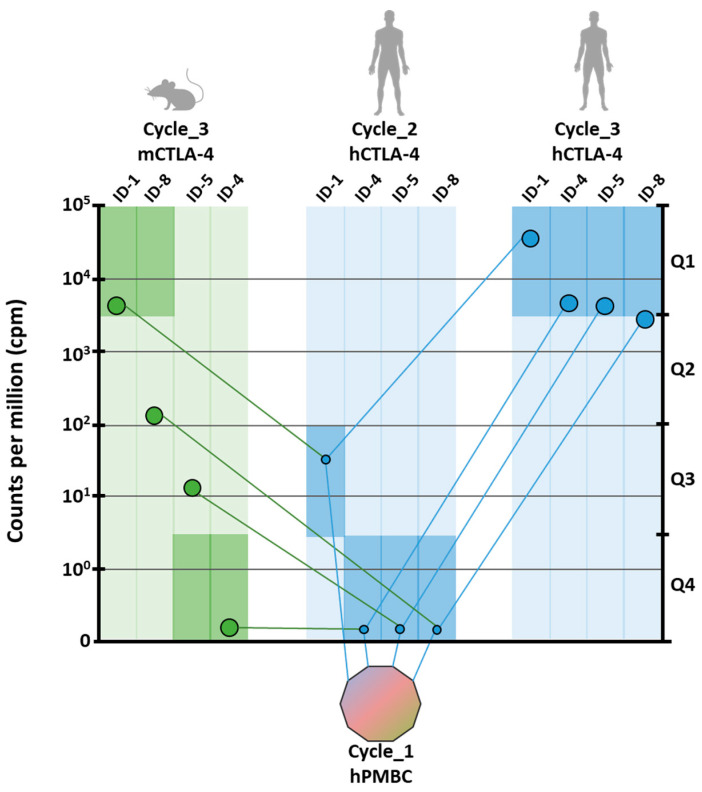
Screening strategy and next generation sequencing data analysis. The screening was carried out starting from the first panning round on hPBMC indicated as colored decagon. The human recombinant cytotoxic T lymphocyte-antigen 4 (CTLA-4) protein was used as bait in the second selection cycle and the relative enrichment of indicated clones was represented as small circles. Human and murine CTLA-4 recombinant proteins were used to perform the third parallel panning rounds. The count per million values (cpm) values for each clone are depicted in the corresponding side of the figure (left side in light green for murine; right side in light blue for human) as large circles. The ranking of ID-1, ID-4, ID-5, and ID-8 clones was also determined according to the belonging quartile (Q1, Q2, Q3, Q4) in each sub-library as indicated by dark green (in murine sub-library) and dark blue (in human sub-library) rectangles.

The strategy used for the analysis of positive clones is shown in Figure 1. Briefly, after the third selection round, the VH region of the scFv clones was extracted from each sub-library by restriction enzyme digestion, rather than by PCR amplification, to preserve the differences in relative representativeness. Three different barcodes were incorporated, respectively, for human-cycle_2, human-cycle_3 and mouse-cycle_3 sub-libraries. The fragments were pooled into a single run of sequencing on MiSeq Illumina platform (San Diego, CA, USA) to obtain at least 1.5 × 10^6^ sequences from each sample (see Section 4 for details).

Joined reads were translated to merge the same paratopes with synonymous nucleotide sequences. The abundance of each encoded protein sequence was normalized within the proper sub-library according to count per million values (cpm), and the sequences without a significant abundance (<10 cpm) were discarded. As recombinant proteins used as baits were fused to the Fc domain, the sequences that were commonly enriched in CTLA-4 and others sub-libraries obtained from previous screenings [38] were considered as Fc binders and were, accordingly, discarded. The best four scFv clones enriched by the end of the third cycle on the human protein were identified as potential binders and named ID-1, ID-4, ID-5, and ID-8 according to their ranking against the human protein (Figure 1). To predict the cross-reactivity to murine CTLA-4, the ranking of ID-1, ID-4, ID-5, and ID-8 was analyzed in the sub-library from the panning performed on mouse protein. Two out of the four clones resulted significantly enriched in the murine sub-library and were respectively ID-1 and ID-8. Interestingly, ID-1 resulted the highest enriched clone in both human and murine sub-libraries, suggesting the recognition of a conserved region of CTLA-4. Although included in the first quartile of murine sub-library, ID-8 ranked in the fiftieth place among murine binders, because of the enrichment of mouse-specific clones (Figure 1). The enrichment of ID-4 and ID-5 clones in the murine sub-library was not significant and predictive for weak or no binding. On the basis of the analysis of parallel sequencing data, ID-1 and ID-8 clones were considered as potential binders for both mouse and human CTLA-4 and were thus selected for additional characterization. To this aim, the corresponding scFvs were rescued from the library by overlapping PCR, and the cDNAs encoding the variable heavy and light regions were used to generate full IgG1 antibodies.

### 2.2. Binding of the Converted Anti-CTLA-4 mAbs to Human and Mouse Lymphocytes and to Purified CTLA-4/Fc Recombinant Protein

The converted monoclonal anti-CTLA-4 antibodies, ID-1 and ID-8, were analyzed to confirm their binding ability to their own specific targets, by both FACS analyses and ELISA assays on hPBMCs and mouse PBMCs. We also used as a control, the converted antibody, named ID-5, which differently from ID-1 and ID-8, was found to be enriched only in the selection on human protein. Unfortunately, ID-4, which could have been another useful control, was not used due to its low expression yield and stability.

**Figure 2:**
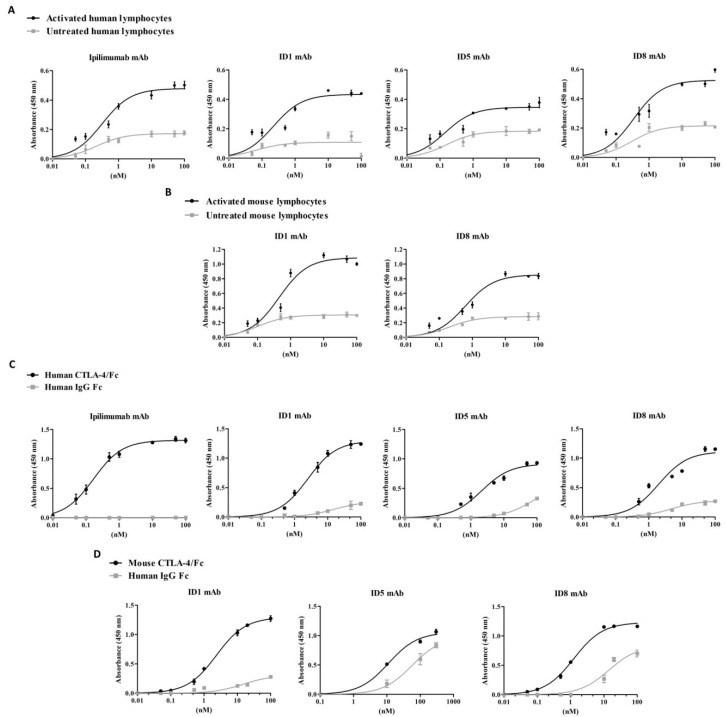
Binding curves of the novel generated anti-CTLA-4 mAbs to human or mouse lymphocytes or to the purified human or mouse recombinant CTLA-4-Fc proteins. Cell ELISA assays on human (**A**) or mouse (**B**) lymphocytes untreated (grey curves) or activated (black curves) for 48–72 h by testing ID-1, ID-8 and ID-5 mAbs at increasing concentrations (0.05–100 nM). Ipilimumab was used as a control. Binding curves of ID-1, ID-8 and ID-5 mAbs to the human (**C**) or mouse (**D**) CTLA-4-Fc purified protein (black curves) or to the corresponding Fc portion (grey curves), used as a negative control. Ipilimumab was used in parallel assays as a control. Error bars depict means ± SD.

The anti-CTLA-4 antibodies were preliminarily tested for their binding to both untreated and activated hPBMCs or mouse PBMCs by FACS analyses at a single concentration (data not shown) and following by ELISA assays by using them at increasing concentrations (Figure 2). As shown in Figure 2A,B, ID-1 and ID-8 mAbs were found able to selectively bind to both the activated human and mouse lymphocytes with higher affinity than that observed on the untreated lymphocytes. On the contrary, ID-5 did not show a highly specific binding to human activated lymphocytes and, as expected, was not cross-reactive for mouse lymphocytes (data not shown). As shown in Table 1, the Kd values for the novel isolated ID-1 and ID-8 antibodies for both activated human and mouse PBMCs, were found to be in the sub-nanomolar range comparable to that observed for Ipilimumab for hPBMCs, used in parallel assays as a positive control. However, accordingly with the selection strategy aimed at obtaining antibodies endowed with mouse-human cross-reactivity, these novel mAbs showed a similar binding affinity for mouse lymphocytes, highlighting different binding properties from Ipilimumab. 

To verify the binding specificity of the novel antibodies, we also tested them by ELISA assays on the recombinant purified proteins. As shown in Figure 2, ID-1 and ID-8 mAbs bound with high affinity and specificity to both the CTLA-4-Fc chimeric proteins, whereas only a poor binding was observed on the Fc, used as a negative control, thus confirming the binding specificity of the two selected antibodies, ID-1 and ID-8, for their targets and the efficiency of the selection strategy designed for both binding selectivity and mouse-human cross-reactivity. The Kd values of ID-1 and ID-8 antibodies for the human CTLA-4 protein are reported in Table 1 for a comparison with that of Ipilimumab, used as a control. ID-5 showed a suboptimal binding for the human chimeric protein and was not significantly cross-reactive for the mouse chimeric protein.

**Figure 3:**
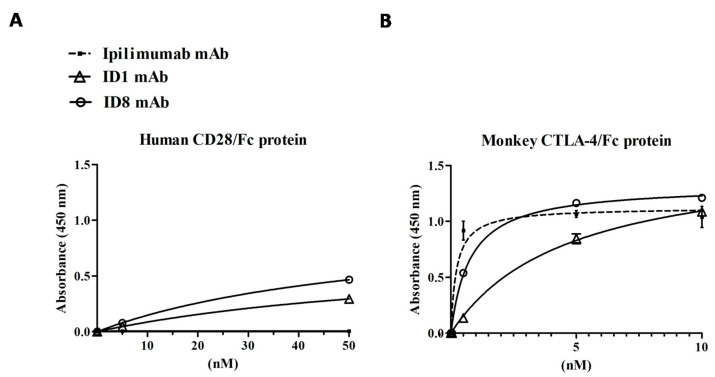
Binding of the novel anti-CTLA-4 mAbs to CD28-Fc and to cynomologous CTLA-4-Fc. Cross-reactivity of ID-1 and ID-8 mAbs to CD28-Fc (**A**) or to monkey CTLA-4-Fc proteins (**B**). Ipilimumab was used in parallel assays as a control.

Considering the homology of CTLA-4 with CD-28 especially in the binding region to their common CD-80 and CD-86 ligands, we also investigated the binding of the novel mAbs to the purified CD-28-Fc chimeric protein. As reported in Figure 3, we found that ID-1 and ID-8 showed only a poor binding to this protein comparable to that observed on the Fc protein, used in parallel assays, thus suggesting that they cannot induce unwanted activation of CD-28. We also tested the cross-reactivity of the anti-CTLA-4 mAbs for the purified cynomolgus CTLA-4-Fc protein by ELISA assays and we found that all the three antibodies were able to recognize this protein accordingly with the high identity of the human and monkey protein sequences (see Figure 3).

### 2.3. Biological Activity of the Novel Anti-CTLA-4 mAbs

Considering the role of CTLA-4 inhibition in potentiating immune responses, we investigated the effects of the novel mAbs on the activation of immune cells. Thus, we firstly measured the ability of ID-1 and ID-8 to induce IL-2 and IFNγ cytokines secretion by unfractionated human and mouse PBMCs in comparison with Ipilimumab and ID-5 mAb, used as controls.

**Figure 4:**
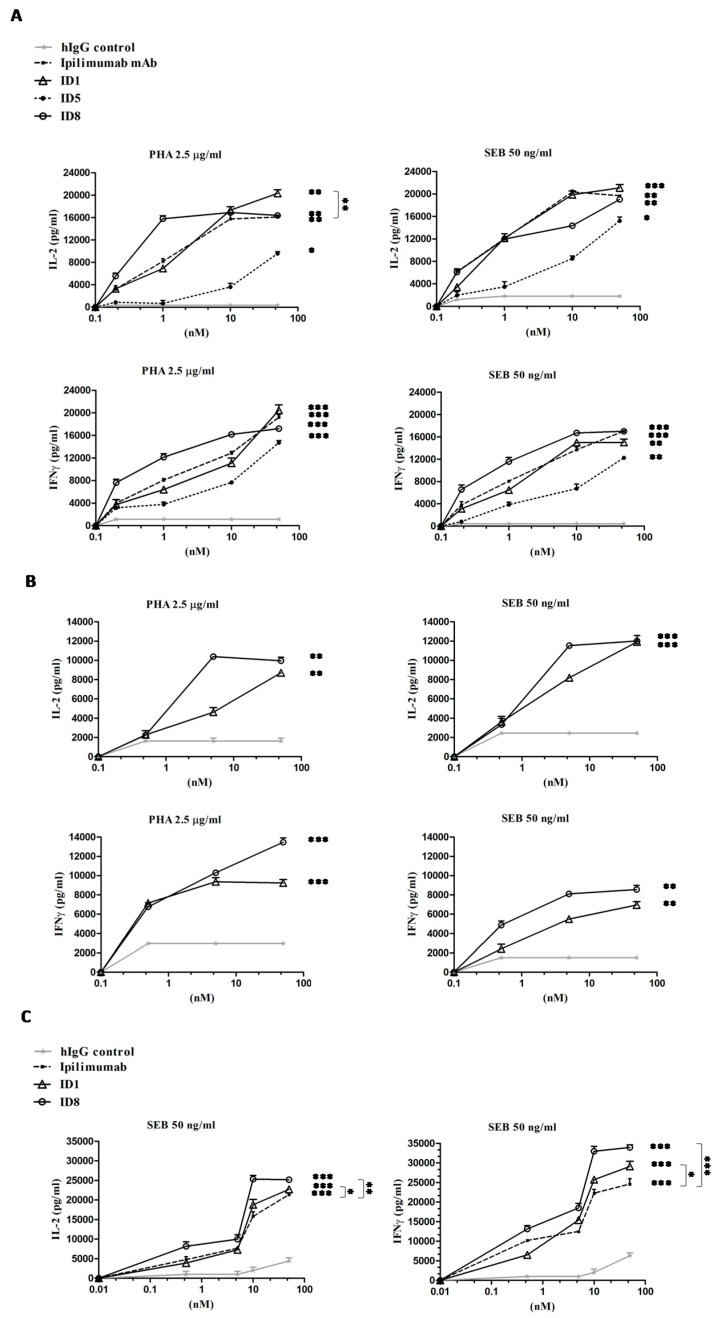
Effects of the novel anti-CTLA-4 mAbs on IL-2 and IFNγ cytokines secretion by stimulated human or mouse PBMC or NK cells. Unfractioned human (**A**) or mouse (**B**) PBMCs or (**C**) natural killer (NK) cells were incubated with ID-1 (triangles), ID-8 (circles), or ID-5 (rhomboids) mAbs at increasing concentrations (0.5–50 nM), in the presence of SEB (50 ng/mL) or PHA (2.5 μg/mL), for 66 h at 37 °C. The levels of cytokine secretion were evaluated by ELISA assays on supernatants of the treated lymphocytes. Ipilimumab or an unrelated IgG antibody was used as positive or negative control, respectively. Error bars were calculated by considering the results obtained by at least three determinations obtained in three independent experiments. Error bars depicted means ± SD. *p*-values for the indicated treatments relative to untreated cells, or to the treatment with Ipilimumab when indicated with vertical bars, are: *** *p* ≤ 0.001; ** *p* < 0.01; * *p* < 0.05.

To this aim, we stimulated the lymphocytes with Staphylococcal Enterotoxin B (SEB) or Phytohaemagglutinin (PHA) used at the concentration of 50 ng/mL and 2.5 μg/mL respectively, in the absence or in the presence of the mAbs used at the concentration range of 0.5–50 nM, for 66 h. As shown in Figure 4A, ID-8 activated the hPBMCs more efficiently than Ipilimumab, whereas ID-1 had a similar activity to that of Ipilimumab. As expected, ID-5 showed a poor activity in the stimulation of hPBMCs compared to that of the other two novel mAbs, thus it has been discarded and not used for further characterization. Instead, both ID-1 and ID-8 antibodies efficiently activated the IL-2 and IFNγ cytokines secretion also by mouse PBMCs (see Figure 4B), confirming their cross-reactivity for the mouse receptor and the possibility to use them on both the species.

Since we recently reported that CTLA-4 is expressed not only by CD3^+^ T cells but also by NK cells [6,40], we decided to further investigate on the effects of the novel mAbs on NK cells, used separately in distinct assays. To this aim, we tested the biological efficacy of ID-1, ID-8, or Ipilimumab, used as a positive control, on a fraction of lymphocytes enriched in NK cells, as described in the Materials and Methods. Once isolated, the cells were stimulated with SEB (50 ng/mL) for 66 h in the absence or in the presence of the mAbs (0.5–50 nM).

The results, shown in Figure 4C, indicate that the novel mAbs activated the NK cell populations slightly stronger than Ipilimumab, by inducing a more efficient secretion of both IL-2 and IFNγ cytokines in particular comparing ID-8 with Ipilimumab. Furthermore, their effects and those of Ipilimumab on NK cells are comparable to those observed on unfractionated hPBMCs, confirming our previous observations [6] on the role of NK cells in the immune responses mediated by anti-CTLA-4 mAbs, such as Ipilimumab.

**Figure 5:**
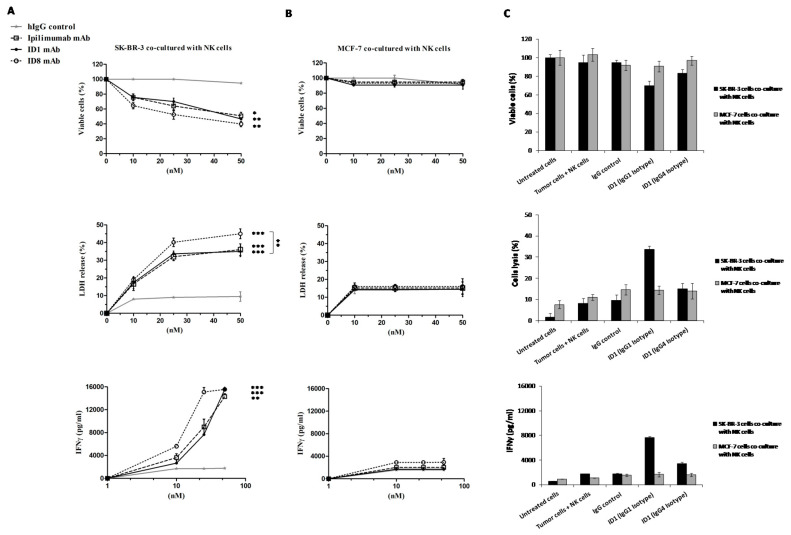
Cytotoxic effects of the novel anti-CTLA-4 mAbs on CTLA-4-positive or -negative tumor cells co-cultured with NK cells. CTLA-4-positive SK-BR-3 (**A**) or CTLA-4-negative MCF-7 (**B**) cells were co-cultured with NK cells (Effector:Target cells ratio 3:1) and incubated, for 24 h in the presence of SEB (50 ng/mL) with ID-1 (full circles) and ID-8 (empty circles) mAbs at increasing concentrations (10–50 nM); Ipilimumab (empty square) and an unrelated IgG (grey curve) antibody were included as controls. In a parallel assay the ID-1 mAb was also tested on both the indicated cell lines in both IgG1 and IgG4 isotypes (**C**). The percentage of viability is expressed as percentage of viable cells with respect to those of untreated cells. Cell lysis was measured by the lactate dehydrogenase (LDH) release in the medium from tumor cells after the incubation with the indicated compounds. The levels of LDH are expressed as percentage of lysis of treated cells with respect to the effects observed in co-cultures of tumor cells with NK cells in the absence of mAbs, used as controls. IFNγ cytokine secretion levels were expressed in pg/mL and measured by ELISA assays performed on cell supernatants as described in Methods. Error bars were calculated by considering the results obtained by at least three determinations obtained in three independent experiments. Error bars depict means ± SD. *p*-values for the indicated treatments relative to untreated cells, or to the treatment with Ipilimumab when indicated with vertical bars, are: *** *p* ≤ 0.001; ** *p* < 0.01; * *p* < 0.05.

On the basis of these promising results, we decided to further investigate these novel interesting effects of Ipilimumab and novel mAbs also on co-cultures of tumor cells with NK cells, in order to test whether the anti-tumor activity is enhanced also in a microenvironment similar to that of in vivo solid tumors. To this aim, CTLA-4-positive SK-BR-3 tumor cells were co-cultured with enriched NK cells (Effector:Target ratio 3:1) and treated for 24 h in the absence or in the presence of ID-1, ID-8, or Ipilimumab used as a control at the same concentrations. As shown in Figure 5, ID-8 induced the death of tumor cells more efficiently than Ipilimumab, by significantly increasing the secretion of IL-2 and IFNγ cytokines by NK cells, and leading to increased cell lysis with a higher lactate dehydrogenase (LDH) release by tumor cells. As expected, both the novel antibodies and Ipilimumab showed no significant effects on MCF-7 tumor cells expressing very low levels of CTLA-4 and used as a negative control in parallel assays. As a further control, we also tested in a parallel assay, the IgG4 isotype of ID-1 for a comparison with the IgG1 isotype (see Figure 5C). As expected, the ID-1 antibody inhibited the cell viability and induced cell lysis more efficiently in the IgG1 isotype due to its ability to induce ADCC, not shared by IgG4, which seems to be essential for the full anti-tumor activity of the anti-CTLA-4 mAb.

**Figure 6:**
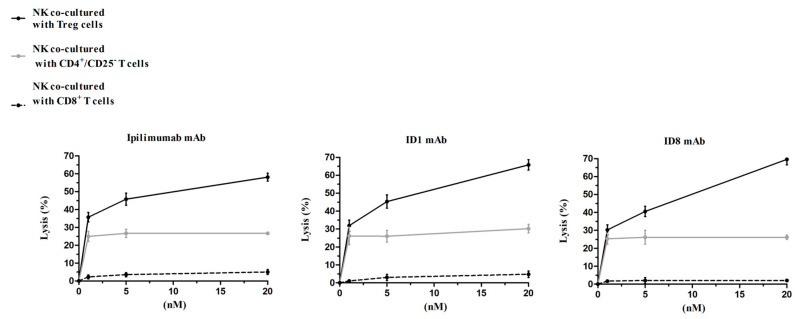
ADCC assays by testing the IgG1 isotype of the novel isolated mAbs on co-cultures of NK with Treg cells or with control CD4^+^/CD25^−^ T cells. NK cells were co-cultured with Treg (black curve), CD8^+^ (dashed curve), or CD4^+^/CD25^−^ (grey curve) T cells (Effector ratio 3:1) in the absence or in the presence of the indicated treatment at increasing concentrations. The effect of ADCC induced by NK cells was measured by LDH release from the indicated T cells populations after 24 h from treatments.

Since many reports in literature [34,35] indicate that Ipilimumab eliminates Treg by inducing ADCC, we tested the novel mAbs for their ability to induce ADCC on this population of T cells, isolated from unfractionated hPBMCs as described in Materials and Methods. Thus, the isolated Treg cells were incubated with NK cells (Effector:Target ratio 3:1) for 24 h in the absence or in the presence of increasing concentrations of ID-1, ID-8, or Ipilimumab used as a positive control. As shown in Figure 6, the novel mAbs show the ability to induce Treg killing by ADCC in a similar fashion to Ipilimumab, thus suggesting that they are effective in killing both tumor cells and inhibitory Treg cells by efficiently activating NK cells. To test whether these effects were selective for Treg cells expressing high levels of CTLA-4, we also tested in parallel assays their effects on CD4^+^ T cell population, previously depleted of Treg cells, and on CD8^+^ T cells. As shown in Figure 6, the three antibodies did not have effects on CD8^+^ T cells and induced only a limited cell lysis on the control CD4^+^/CD25^−^ T cells, thus confirming their specific ADCC on Treg cells.

These results strongly suggest that the novel mAbs could have a therapeutic potential similar to clinically effective anti-CTLA-4 mAbs, reported to cause tumor rejection by mechanisms that are dependent on the host Fc receptor [41]. Indeed, Treg depletion and Fc receptor-dependent tumor rejection have been recently reported to play a major role for Ipilimumab and other anti-CTLA-4 antibodies with respect to checkpoint blockade dependent on B7-CTLA-4 interactions [42].

### 2.4. Cell Growth Inhibitory Effects of the Novel Anti-CTLA-4 mAbs on Tumor Cells

Recent reports by our and other groups [6] have evidenced the expression of CTLA-4 also on the surface of tumor cells, thus we investigated the effects of the human anti-CTLA-4 mAbs on cell lines expressing different levels of this antigen to verify whether they show also a direct anti-tumor cell activity independent from the immune response.

**Figure 7:**
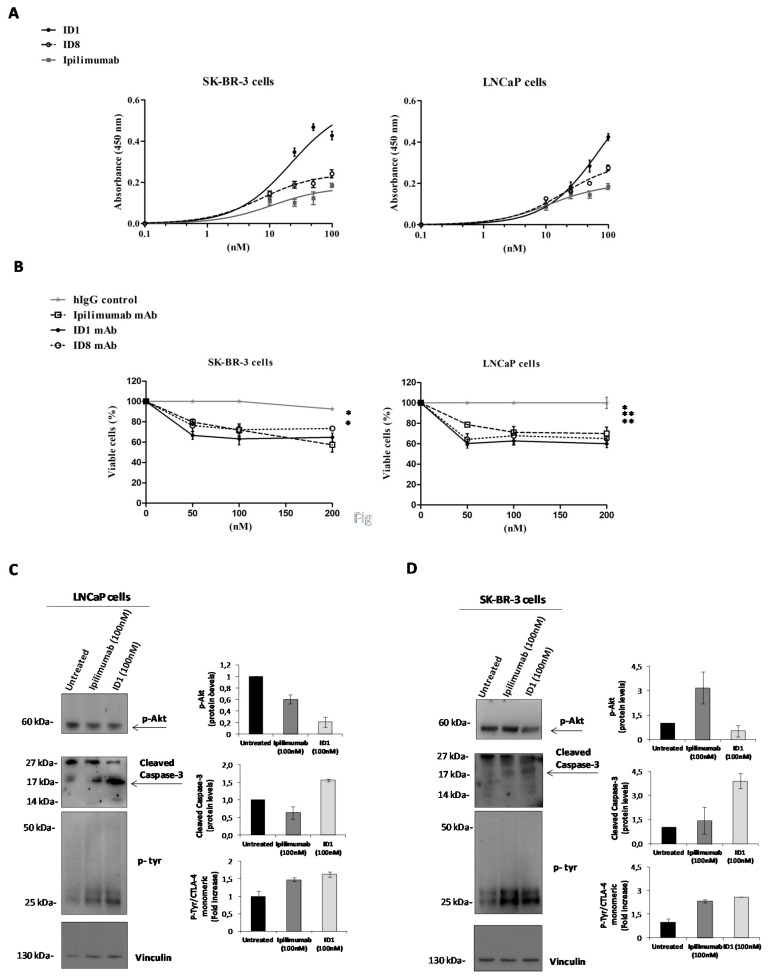
Binding of the novel isolated anti-CTLA-4 mAbs to tumor cells and their effects on the growth and on intracellular pathways downstream CTLA-4. (**A**) Binding curves of ID-1, ID-8, or Ipilimumab mAbs tested by ELISA assays at increasing concentrations on SK-BR-3 and LNCaP tumor cells. (**B**) Anti-tumor effects of ID-1, ID-8 or Ipilimumab mAbs on SK-BR-3 and LNCaP cells treated with increasing concentrations for 72 h at 37 °C. The percentage of viable cells is expressed with respect to untreated cells. Cells treated with an unrelated IgG are the negative control. Error bars depict means ± SD. *p*-values for the indicated treatments relative to untreated cells are: ** *p* < 0.01; * *p* < 0.05. Western blotting analyses with the antibodies specific for the indicated proteins of cell extracts from LNCaP (**C**) and SK-BR-3 (**D**) tumor cells, treated in the absence or in the presence of Ipilimumab or ID-1 mAb for 72 h. Protein levels are expressed as fold increase with respect to untreated cells and normalized to vinculin.

Firstly, we analyzed the expression of CTLA-4 on these cells by measuring the corresponding binding of the antibodies by ELISA assays. As shown in Figure 7A, all three of the anti-CTLA-4 mAbs, ID-1, ID8, and Ipilimumab, bound to SK-BR-3 and LNCaP cells, thus confirming that they express CTLA-4, whereas no significant binding was observed on MCF-7 cells, used as a negative control (data not shown). Interestingly, ID-1 showed a higher binding affinity than Ipilimumab for the CTLA-4 positive tumor cells.

We then tested the effects of the novel mAbs in comparison with Ipilimumab on tumor cell viability of SK-BR-3 and LNCaP cells when incubated at increasing concentrations for 72 h. As shown in Figure 7B, the three antibodies inhibited the growth of both CTLA-4-positive tumor cells also independently from the immune system. As expected, the novel antibodies and Ipilimumab showed no significant effects on MCF-7 tumor cells expressing very low levels of the two antigens and thus used as a negative control (data not shown).

In order to investigate the molecular basis of these effects, we analyzed the intracellular pathways downstream CTLA-4 in SK-BR-3 and LNCaP treated cells by Western blotting analyses of cell extracts with anti-CTLA-4, anti-pTyr, anti-pAkt, and anti-caspase 3 antibodies. As shown in Figure 7, both Ipilimumab and ID1 induce a marked phosphorylation of the monomeric form (25 kDa) of CTLA-4 likely acting on downstream intracellular pathways, but the novel mAb inhibits the phosphorylation of Akt and induces the cleavage of caspase 3 (arrows indicate the corresponding bands in the blots) more efficiently than Ipilimumab. We cannot exclude that Ipilimumab affects other unknown downstream pathways leading to the cell growth inhibition observed in Figure 7B.

**Figure 8:**
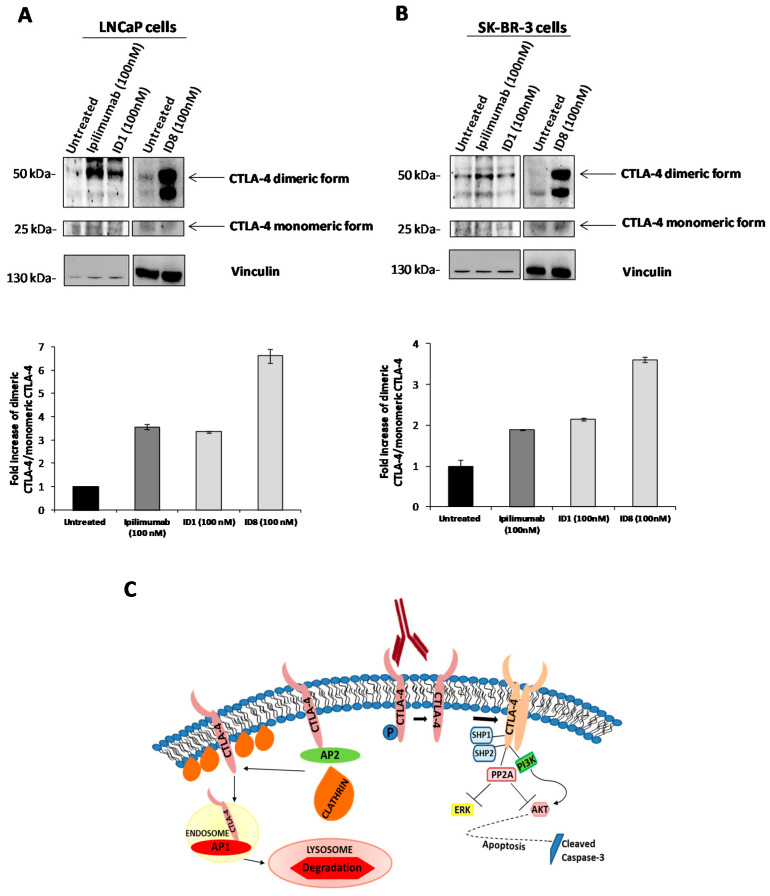
Effects of anti-CTLA-4 mAbs on monomeric or dimeric CTLA-4 form in tumor cells. Western blotting analyses with the antibodies specific for CTLA-4 protein of cell extracts from LNCaP (**A**) and SK-BR-3 (**B**) tumor cells, treated in the absence or in the presence of Ipilimumab, ID-1 or ID-8 mAb for 72 h. Protein levels are expressed as fold increase of dimeric vs. monomeric form (see arrows) with respect to untreated cells and normalized to vinculin. (**C**) Model proposed to explain the effects of the novel mAbs on CTLA-4 expressed on tumor cells and its downstream intracellular pathways.

These results suggest that the novel mAb directly affects the CTLA-4 function on tumor cells by inhibiting the downstream survival pathways, such as that of PI3K, and by inducing apoptosis by downregulating Akt, thus acting as an agonistic antibody on tumor cells. Indeed, ID-1 has a similar effect of apoptosis induction exerted on the CD-80 and CD-86 ligands on these tumor cells (data not shown), accordingly with previous results reported in literature [15]. Similar results were obtained also with ID8 (data not shown), which in addition induces a more marked homodimerization of CTLA-4 (Figure 8, the arrows indicate the corresponding dimeric and monomeric forms in the blots), thus increasing the level on the cell surface of the receptor, in line with the hyperphosphorylation of the monomeric form of CTLA-4, which is required to avoid its internalization and the subsequent degradation [20,21,23]. Furthermore, the dimeric form of CTLA-4 is able to recruit, through its cytoplasmatic tail not only PI3K but also phosphatases, such as PP2A, which could inhibit ERK (see Figure 8), as previously reported in literature [19,26,27,43].

**Figure 9:**
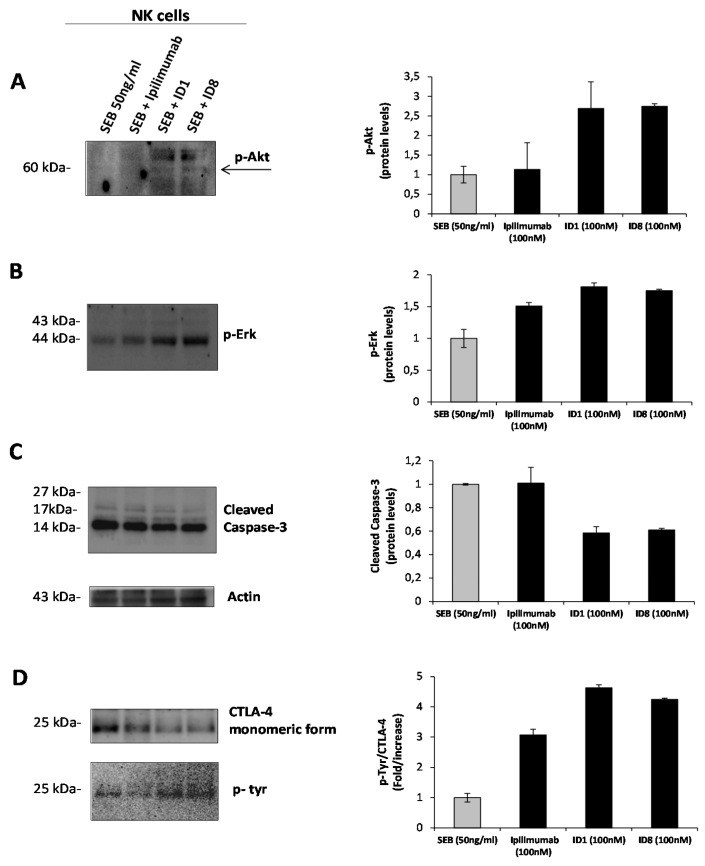
Effects of the novel anti-CTLA-4 mAbs on intracellular pathways downstream CTLA-4 in NK cells. NK cells, treated with ID-1, ID-8, or Ipilimumab, under stimulation with SEB (50 ng/mL) for 66 h. Western blotting analyses of cell extracts from NK cells, treated as indicated, with the antibodies specific p-Akt (**A**), p-Erk (**B**), caspase 3 and actin (**C**) or CTLA-4 and p-tyr (**D**). Protein levels are expressed as fold increase with respect to untreated cells and normalized to actin.

As a further control, we analyzed in parallel assays the intracellular pathways downstream CTLA-4 in NK activated cells treated in the absence or in the presence of the anti-CTLA-4 mAbs for 66 h, by Western blotting analyses of cell extracts with anti-CTLA-4, anti-pTyr, anti-pErk, anti-pAkt, and anti-caspase 3 antibodies. As shown in Figure 9, as expected, we found that the three antibodies show opposite effects on NK cells, accordingly to their opposite effects on the proliferation of these cells, as they induce the phosphorylation of Erk and Akt, and inhibit the cleavage of caspase, thus indicating that on NK cells they activate the proliferation and survival pathways and inhibit apoptosis. The novel ID-1 and ID-8 mAbs induce a more marked phosphorylation of the monomeric form (25 kDa) of CTLA-4 also on NK cells with respect to Ipilimumab, thus suggesting that this event could likely induce the activation of the pathways downstream CTLA-4. Indeed, Ipilimumab did not affect the level of pAkt and cleaved caspase, showing only slight effects on the activation of Erk. These results suggest that the novel antibodies could have a different mechanism of action with respect to Ipilimumab and shed light on the different roles played by CTLA-4 on different cell populations, such as immune and tumor cells, demonstrating its capacity of inducing either proliferation or apoptosis, respectively.

To further test the formulated hypothesis of the different mechanism of action of ID-1 and ID-8 from that of Ipilimumab, we investigated on their potential antagonistic activity by measuring their ability to interfere in the binding of CTLA-4 with its ligands CD-80 and CD-86 in competitive ELISA assays (see Supplementary Figure S1). We found that Ipilimumab was more efficient than the novel antibodies in inhibiting the interaction of CTLA-4 with their ligands, thus confirming that its well-known antagonistic effect is higher than that of ID-1 and ID-8, that instead showed only a partial inhibition (40–50%) of the binding of CTLA-4 with their ligands (see Supplementary Figure S1). Furthermore, the novel mAbs seem to recognize a different epitope of CTLA-4 from that of Ipilimumab, as evidenced from competitive ELISA assays performed by measuring the binding to immobilized CTLA-4 of biotinylated Ipilimumab in the absence or presence of saturating concentrations of unlabeled ID-1 and ID-8 mAbs (Supplementary Figure S2). Indeed, the binding curves of biotinylated Ipilimumab in the absence or in the presence of ID-1 or ID-8 mAbs are superimposable, thus suggesting that the novel mAbs do not interfere with Ipilimumab in the interactions with CTLA-4.

To confirm the different behavior of ID-1 and ID-8 from that of Ipilimumab in the interference of receptor-ligand binding we used also a different approach with a cell-based bioluminescent assay. In this assay effector Jurkat T cells expressing human CTLA-4 and CD28 coupled with a luciferase reporter driven by a NFAT response element (NFAT-RE) were co-cultured with APC Raji cells expressing human CD80 and CD86 and an engineered cell surface protein designed to activate the TCRs in an antigen-independent manner. Ipilimumab efficiently inhibited the interaction of CTLA-4 with their ligands, thus activating the intracellular pathway downstream CD28, as indicated by the increase in luminescence values whereas ID-1 and ID-8 were much less effective than Ipilimumab in this assay (Supplementary Figure S3), suggesting that they only slightly affected this interaction in line with the results obtained by the above mentioned ELISA assays. Unfortunately, it was not possible to analyze the effects of the mAbs on the other pathways downstream CTLA-4 in CD8^+^ T cells, as shown before for tumor and NK cells, since the isolated CD8+ cells were not significantly stimulated with SEB (under the conditions used for activating NK cells) either in the absence or in the presence of the mAbs (Supplementary Figure S3).

Altogether these results suggest that the novel anti-CTLA-4 mAbs could have a different mechanism of action from that of Ipilimumab and could be devoid of the immune related adverse side effects correlated with its antagonist activity.

### 2.5. Pro-Inflammatory Effects of Ipilimumab and the Novel Anti-CTLA-4 mAbs

**Figure 10:**
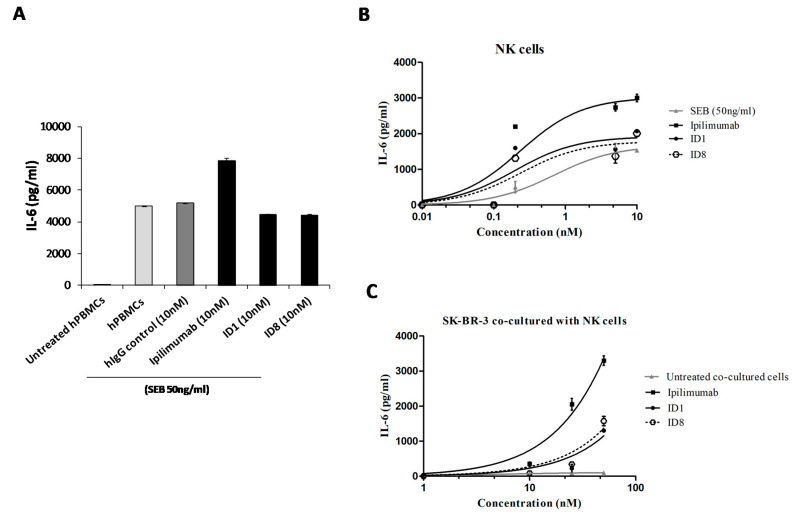
Pro-inflammatory IL-6 secreted after anti-CTLA-4 mAbs treatments. Interleukin-6 production by hPBMCs (**A**), or NK cells (**B**) treated with ID-1, ID-8 or Ipilimumab mAb for 66 h with SEB or co-cultured for 24 h in the presence of SKBR3 tumor cells (**C**). Untreated cells or cells treated with an unrelated IgG were used as negative controls. Details of the experiments were described in Materials and Methods.

To test the potential adverse side effects of the anti-CTLA-4 mAbs, we measured the secretion of the pro-inflammatory cytokine IL6 after treatment of hPBMCs or NK cells with the novel mAbs. Interleukin 6 is a key regulator of immune-related reactions involved in inflammatory processes, such as autoimmune diseases or anticancer-induced cardiotoxicity. We found that Ipilimumab increased cellular secretion of Interleukin 6 in the supernatant of both Lymphocytes either activated with SEB or co-cultured with SKBR3 tumor cells, as shown in Figure 10. The treatment with the novel mAbs show instead only low levels of this interleukin, even though they induce secretion of IL2 and IFNγ at concentrations comparable to those of Ipilimumab (Figure 4). These encouraging results suggest that the novel mAbs could be devoid of pro-inflammatory side effects and confirm that they have a different mechanism of action from that of Ipilimumab.

## 3. Discussion

CTLA-4 has been considered for a long time an immune checkpoint expressed exclusively on T cells and involved in the inhibition of the signaling downstream the T-cell co-stimulatory CD28 receptor, as it competes for the binding to its CD80 and CD86 ligands by showing higher affinity [8]. However, its expression on other cell populations, such as tumor or NK cells lacking CD28 [6], suggests that it could have other unrevealed roles on different cell populations. Indeed, other possible mechanisms of action of this receptor could involve its intracellular recruitment of phosphatases and/or signaling pathways through PI3K, as it contains two tyrosine residues in the C-terminal tail, that once phosphorylated, can become docking sites for intracellular adaptor proteins [19,24,25,26].

Anti-CTLA-4 antibodies, such as Ipilimumab, can induce successful anti-cancer therapeutic effects especially in melanoma patients [29,30]. However, some of its limitations are represented by its significant immunotherapy-related adverse effects (irAEs) that have been difficult to predict in pre-clinical models due to its low cross-reactivity for the mouse protein.

In this study, we isolated two novel human anti-CTLA-4 mAbs, selected by phage display on live activated lymphocytes and purified recombinant protein by using an innovative strategy of high throughput screening aimed at identifying antibody sequences commonly enriched by two parallel selections on human and mouse CTLA-4 protein. We found that these two identified antibodies, called ID-1 and ID-8, bind with high affinity to both human and murine purified proteins and lymphocytes showing nanomolar or sub-nanomolar Kd values, thus confirming the efficiency of the selection strategy.

More importantly, the novel selected antibodies were able to induce the activation of both human and mouse unfractionated PBMCs with a similar potency of Ipilimumab by strongly increasing the secretion of IL-2 and IFNγ cytokines.

Interestingly, we found that ID-8 stimulated NK cells more efficiently than Ipilimumab and showed a more potent cytotoxic activity against tumor target cells by an efficient redirection of NK lymphocytes against cancer cells when co-cultured, thus strongly enhancing the ADCC effects. We tested these effects also on co-cultures of NK cells and Treg cells and we demonstrated that both the novel antibodies were as effective as Ipilimumab in killing Treg cells by ADCC with no significant effects on the other CD4^+^ T cells.

Finally, when tested on cultures of tumor cells, the novel antibodies were found able to inhibit cell growth with a different mechanism of action from that of Ipilimumab. In particular, the novel ID-1 and ID-8 mAbs induced a marked phosphorylation of CTLA-4, inhibited the phosphorylation of Akt and induced caspase activation differently from Ipilimumab in the two CTLA-4-positive cell lines tested. These results suggest that the novel mAbs directly affect the CTLA-4 function on tumor cells by inhibiting the downstream survival pathways, such as that of PI3K, and by inducing apoptosis in a similar fashion to CD-80 and CD-86 ligands, that induced apoptosis by caspase 3 activation, as also previously reported in literature [15,16,18]. Furthermore, the novel mAbs induced a marked homodimerization of CTLA-4, thus increasing the level on the cell surface of the receptor, in line with the hyperphosphorylation of the monomeric form of CTLA-4, required to avoid its internalization and the subsequent degradation [20,21,23]. Interestingly all the three antibodies showed opposite effects on NK cells, accordingly to their opposite effects on the proliferation of these cells, as they induce the phosphorylation of Erk and Akt, and inhibit the cleavage of caspase, thus indicating that on NK cells they activate the proliferation and survival pathways and inhibit apoptosis. These results suggest for the first time that more attention should be paid to the role of CTLA-4 in different lymphocytes subsets and cell populations, as it can regulate different downstream pathways and consequent effects.

When tested for their ability to interfere in the binding of CTLA-4 to its ligands CD-80 and CD-86 in competitive ELISA assays, ID-1 and ID-8 instead showed only a partial inhibition (40–50%) of the binding of CTLA-4 with their ligands differently from Ipilimumab that displayed its full antagonistic activity, thus suggesting that the novel anti-CTLA-4 mAbs could have a different mechanism of action from that of Ipilimumab.

Since it has been proposed that Checkpoint blockade may be unnecessary for the effector function of anti-CTLA-4 antibodies [44] as they induce tumor rejection mainly by selective depletion of Tregs in the tumors rather than blockade of B7-CTLA-4 interaction in lymphoid organs, the partial efficiency of the novel mAbs with respect to Ipilimumab in this blockade activity should not affect their anti-tumor activity, whereas on the contrary it could represent an advantage for reducing the unwanted immune response adverse events. Furthermore, since the novel antibodies do not induce the degradation of CTLA-4, they could provide safe and more efficient anti-tumor responses, differently from those antibodies that trigger degradation of CTLA-4 increasing the toxicity and reducing anti-tumor efficacy, as previously reported [39]. Indeed, an important feature for novel generation anti-CTLA-4 antibodies is the uncoupling of their therapeutic effects from irAEs. Up to date the immunotherapeutic effects have been achieved through the antagonistic activity of the anti-CTLA-4 antibodies, i.e., blocking the checkpoint that can prevent autoimmunity and immune responses against cancer [45], thus making it difficult to achieve cancer immunity without irAEs.

The novel antibodies, devoid of the antagonistic activity but still endowed with potent effects on NK cells, could provide efficient anti-tumor effects through selective tumor and Treg cells killing by ADCC without the irAEs associated with the antagonistic activity of Ipilimumab. This aspect could be even more important in case of combinatorial treatments such as the successful combination, previously reported [46,47], of anti-PD-1 and anti-CTLA-4 antibodies resulting in a longer progression-free survival and a higher rate of response but leading to increased autoimmune reactions representing the major toxic effects of this class of therapeutic antibodies. Further studies are needed to carefully monitor auto-immune side effects, potentially induced by these novel antibodies; however, their lack of antagonistic activity could allow for reducing eventual unwanted side effects even when combined with other immunomodulatory mAbs.

Furthermore, the mouse–human cross-reactivity of the novel antibodies confirms that their binding properties are different from those of Ipilimumab and make them valuable tools for future studies in mouse models, unaffordable before with Ipilimumab due to its low cross-reactivity for mouse CTLA-4.

## 4. Materials and Methods

### 4.1. Cell Lines

SK-BR-3 and LNCaP cells were cultured in RPMI 1640 (Gibco BRL, Life Technologies, Paisley, UK), whereas MCF-7 cells were cultured in Modified Eagle’s Medium (MEM, Gibco, Life Technologies, Grand Island, NE, USA). All the three cell lines, obtained from ATCC (Rockville, MD, USA), were grown in humidified atmosphere containing 5% CO₂ at 37 °C, in media supplemented with 10% heat-inactivated fetal bovine serum (FBS, Sigma-Aldrich, St. Louis, MO, USA), 50 U/mL penicillin, 50 μg/mL streptomycin and 2 nM L-glutamine (all from Gibco, Thermo Fisher Scientific, Paisley, UK).

### 4.2. Isolation of Human and Mouse Peripheral Blood Mononuclear Cells

Human and mouse PBMCs, isolated and frozen following the manufacturer’s instructions as previously reported [38,48], were thawed out by using the RPMI medium supplemented with 2 nM L-glutamine, 1× CTLWash Supplement 10× (Cellular Technology Limited, Shaker Heights, OH, USA) and 100 U/mL Benzonase (Merck Millipore, Burlington, USA). PBMCs were grown in R10 medium made up of RPMI 1640 medium, supplemented as described above, with the addition of 10 mM HEPES, and 50 mM β-Mercaptoethanol (both from Gibco Thermo Fisher Scientific, Paisley, Scotland, UK) for mouse lymphocytes. After an overnight resting at 37 °C, the cells were counted by using the Muse cell analyzer (Merck Millipore, 0500-3115, Darmdstadt, Germany) and used for assays.

### 4.3. Isolation of NK Cells

Human NK cells were isolated from hPBMCs, as previously reported [6]. Briefly, they were obtained from unfractioned hPBMCs by using the NK Cell Isolation Kit (MACS, Miltenyi Biotec, Bergisch Gladbach, Germany), by following the manufacturer’s guidelines, and counted by using a Muse cell analyzer and cultured in R10 medium at 37 °C.

### 4.4. Isolation of Treg Cells

CD4^+^/CD25^+^ regulatory T cells were isolated from unfractioned hPBMCs by using the Dynabeads Regulatory CD4^+^CD25^+^ T Cell Kit (Invitrogen by Thermo Fisher Scientific, Baltics UAB, Lithuania), following the manufacturer’s instructions. Briefly, starting from PBMCs the non CD4^+^ cells were labeled with the antibody mix provided by the kit and then incubated with the depletion MyOne dynabeads for their removal. The flow through containing the CD4^+^ T cells was incubated with the dynabeads CD25, to capture the CD4^+^/CD25^+^ T cells, and placed on a magnetic stand to remove the supernatant containing the CD4^+^/CD25^−^. The bound cells were eluted by an incubation with the DETACH-BEAD reagent after extensive washes, and collected by placing the sample on a magnetic separator in order to remove the Dynabeads CD25. The supernatant containing the CD4^+^/CD25^+^ isolated Treg cells was centrifuged at 350× *g* for 8 min and the cell pellet was then resuspended in R10 medium, before counting the cells by using a Muse cell analyzer.

### 4.5. Antibodies and Recombinant Proteins

The following antibodies were used: anti-CTLA-4 mAb Ipilimumab (Yervoy, Bristol Myers Squibb, NY, USA); commercial Human CTLA-4 Antibody (R & D Systems, Minneapolis, MN, USA); anti-phospho44/42 MAPK (indicated as pErk); anti-phospho-(Ser/Thr) Akt; anti-Cleaved Caspase-3 polyclonal antibody antibodies (all from Cell Signaling Technology, Danvers, MA, USA); anti-vinculin; anti-p-Tyr polyclonal antibodies (both from Santa Cruz Biotechnology, Inc. Dallas, Texas USA; anti-actin antibody (Sigma-Aldrich, Darmdstadt, Germany); anti-His HRP conjugated mAb (Qiagen, Hilden, Germany); anti-human IgG (H+L) HRP conjugate antibody (Promega, Madison, WI, USA); anti-human IgG (Fab’)2 goat monoclonal antibody (Abcam, Cambridge, UK, ab98535).

The following recombinant proteins were used: Recombinant Human CTLA-4 Fc Chimera; Recombinant Human B7-1/CD80 Fc Chimera Protein; Recombinant Human B7-2/CD86 Fc Chimera Protein; Recombinant Human CD28 Fc protein; Recombinant Cynomolgus Monkey CTLA-4 Fc Chimera Protein; Recombinant Human IgG1 Fc Protein (all from R & D Systems, Minneapolis, MN, USA); Human CTLA-4 Protein (His & Fc Tag); CTLA-4 Protein, Mouse, Recombinant (Fc Tag) (both from Sino Biological, Wayne, PA, USA).

### 4.6. Selection of scFv-Page Clones

Phagemid particles, recovered from the cells infected with the library by using the M13-K07 helper phage (Invitrogen, Thermo Fisher Scientific, Carlsbad, CA 92008, USA) were isolated by precipitation with PEG (10^13^ cfu), as previously described [38,49,50]. In a first round the phages were incubated with human PBMCs, previously activated with Dynabeads Human T-Activator CD3/CD28 (ThermoFisher Scientific, Baltics UAB, Lithuania) for 96 h. The recovered phages were amplified by infecting *E. coli* TG1 cells to prepare phages for the following second round performed on coated Recombinant Human CTLA-4 Fc protein (20 μg/mL), followed by two parallel rounds of selection on coated Recombinant Human or Mouse CTLA-4 Fc proteins. Before each round of selection on the chimeric proteins, the phages were submitted to two subsequent rounds of negative selection on Recombinant Human IgG1 Fc Protein to remove phage-scFv recognizing the Fc domain, as previously described [38].

### 4.7. Preparation of DNA Fragments and Generation of Libraries for High-Throughput Screening

Phagemid double-strand DNAs containing the scFvs were prepared by Endo free Plasmid Maxi Kit (Qiagen, 12362) from the three sub-libraries. The variable heavy chains were extracted in a two-step restriction process by BamHI (R3136) and HindIII (R3104) followed by NcoI (R3193) and XhoI (R0146) (New England Biolabs, Ipswich, Massachusetts, USA) as previously reported. Libraries were prepared by TruSeq ChIP sample prep kit (Illumina, 15,023,092) and sequenced to a final concentration of 10 pM with 2 × 300 nt SBS kit v3 on an Illumina MiSeq apparatus [51]. The sequencing was performed at the Center for Translational Genomics and Bioinformatics, Hospital San Raffaele, Milano, Italy. Paired end 2 × 300 sequencing was performed and FastQC software was used to examine quality of fastq files. Reads were joined using fastq-join and Fastqc was used for quality checks on joined sequences. Raw counts were normalized to the total number of counts within each sub-library, obtaining counts per million values. Joined sequences were translated considering the correct open reading frame.

### 4.8. scFv Reconstitution and Antibodies Production

The full length sequences of scFvs of interest were isolated from human_Cycle3 by overlapping PCR. Briefly, using clone-specific primers designed within the heavy CDR3 region, VH and VL fragments were obtained separately in two PCR reactions by Phusion High-Fidelity DNA Polymerase (Thermo Fisher Scientific, F530S, Waltham, Massachusetts, USA). The two obtained fragments were overlapped by CDR3 sequence and extended to get the full scFvs [51]. The scFvs of interest were converted into whole human IgG1 and IgG4 antibodies by cloning the corresponding VH and VL cDNAs in SINEUP-competent, Fc encoding vectors by In-Fusion HD cloning kit (Clontech Laboratories, 639,692. Mountain View, California, USA). PEUVL4.2_SA was used to subclone the VL sequences. PEUVH1.2 and PEUVH8.2 were respectively used to generate the selected antibodies in IgG1 and IgG4 isotypes [52]. The vectors encoding the heavy and light chains of interest were prepared with an endotoxin-free system (EndoFree Plasmid Maxi Kit, Qiagen, 12,362) and were co-transfected in HEK293EBNA SINEUP (HEK293ES_1) cells by using Lipofectamine Transfection Reagent (Life Technologies, Inc. 11,668,019. Carlsbad, California, USA) and grown up for about 10 days at 37 °C in chemical defined CD CHO medium (Gibco, Life Technologies, Inc. 10,743,029) complemented with 5 mL of L-glutamine 200 mM (Gibco, Life Technologies, A2916801), 5 mL of Penicillin-Streptomicyn 10,000 U/mL (Sigma-Aldirch, P0781) in 150 mm Corning^®^ tissue-culture treated culture dishes. The antibodies were purified from the conditioned media by using Protein A HP SpinTrap30 (GE Healthcare Life Sciences, 28–9031–32. Chicago, Illinois, USA). The quality of antibodies preparation was evaluated by SDS-PAGE NuPAGE™ 4–12% Bis-Tris Protein Gels (Thermo Fisher Scientific, NP0321BOX, Waltham, Massachusetts, USA) followed by Coomassie blue staining (Biorad, 1,610,786. Hercules, California, USA). Purified antibodies were desalted and buffer-exchanged by PD-10 Columns (GE Healthcare Life Sciences, 17085101). The antibody preparations were sterilized by filtration with 0.22 µm durapore hydrophilic filters (Millipore, SLGS033SS. Burlington, Massachusetts, USA) and stored in aliquots at −80 °C.

### 4.9. ELISA Assays

ELISA assays were carried out on both lymphocytes and recombinant proteins to test the binding ability of the novel anti-CTLA-4 mAbs. To measure the ability of the mAbs to bind to receptors displayed on the cell surface, human or mouse lymphocytes (4 × 10^5^ cells/well) previously activated with anti-CD3/CD28 beads for 48–72 h, were plated on round-bottom 96-well plates and incubated with the mAbs in PBS/Milk 2.5% buffer solution for 2 h at Room Temperature, by gently shaking. After extensive washes with PBS 1×, the plates were incubated with an anti-human IgG (H+L) HRP-conjugated antibody for 1 h at room temperature.

The binding ability of the mAbs was also tested on the purified recombinant human or mouse CTLA-4/Fc, human CD28/Fc, Monkey CTLA-4/Fc chimeric proteins or the Fc portion used as a negative control in parallel assays. After coating on NuncTM flat-bottom 96-well plates at a concentration of 5 μg/mL and blocking with PBS/milk 5% at 37 °C for 1 h, the immobilized chimeric proteins were incubated with the indicated mAbs in PBS/milk 2.5% buffer solution for 2 h at room temperature. After extensive washes with PBS1× solution, plates were incubated with HRP-conjugated anti-human IgG (Fab’)2 goat monoclonal antibody in PBS/milk 2,5% buffer solution for 1 h at room temperature. Then, the plates were washed again with PBS 1× and incubated with TMB (Sigma-Aldrich, St. Louise, USA) reagent for 10 min, before quenching with an equal volume of 1 N HCl. Absorbance at 450 nm was measured by the Envision plate reader (Perkin Elmer, 2102, San Diego, CA, USA). The Kd values were calculated by elaboration of ELISA binding curve analyses by Prism (GraphPad Prism 5) tool, following the model previously described [38].

### 4.10. Competitive ELISA Assays

In order to investigate the ability of the novel isolated ID-1 and ID-8 mAbs to interfere in the CD80-CTLA-4 or CD86-CTLA-4 interactions, competitive ELISA assays were performed by testing the binding of CD-80 or CD-86 His-tagged-ligands to the chimeric CTLA-4/Fc protein or PBMCs in the absence or in the presence of excess of the indicated mAbs. To this aim, the CTLA-4-Fc protein was coated on NuncTM flat-bottom 96-well plates at a concentration of 500 ng/mL. After blocking in PBS/milk (5%) buffer solution for 1 h at 37 °C, the coated plates were pre-incubated with an excess of the selected mAbs (10:1 M/M ratio) for 2 h at room temperature in PBS/Milk 2.5% buffer solution. Then, CD80 or CD86 ligands were added to the plates (1:1 M/M ratio) and incubated for 2 h at room temperature. After extensive washes, the anti-His HRP-conjugated antibody was added to the plate for 1 h at room temperature in PBS/Milk 2.5% buffer solution for the detection of the bound ligands. After extensive washes, the plates were incubated with TMB reagent for 10 min, before quenching with an equal volume of 1 N HCl. Absorbance at 450 nm was measured by the Envision plate reader.

To investigate whether the novel isolated anti-CTLA-4 mAbs recognize different epitopes from that recognized by Ipilimumab, competitive ELISA assays were performed on plates coated with CTLA-4/Fc protein (5 μg/mL) by testing increasing concentrations of the biotinylated Ipilimumab mAb to CTLA-4 in the absence or in the presence of saturating concentrations (400 nM) of ID-1 or ID-8 mAbs. After extensive washes, the biotinylated Ipilimumab mAb was detected with HRP-conjugated Streptavidin antibody. The plates were then washed again and analyzed as described above.

### 4.11. Cytotoxicity and Cell Growth Inhibition Assays

To test the effects of the novel anti-CTLA-4 mAbs on tumor cells growth, CTLA-4-positive SK-BR-3 cells (1.5 × 10⁴ cells/well) or CTLA-4-negative MCF-7 (10 × 10^3^ cells/well) were plated in 96-well flat-bottom plates and incubated for 16 h at 37 °C. Then, enriched NK cell population was added (Effector: Target cell ratio 3:1) to tumor cells and incubated in the absence or in the presence of ID-1, ID-8 or Ipilimumab mAbs (IgG1 isotype), at increasing concentrations (10–50 nM) for 24 h at 37 °C. ID-1 mAb was also tested in IgG4 isotype at the concentration of 25 nM, as a control. After treatments, the supernatants of the co-cultures were collected by centrifugation at 1200 rpm for 10 min, tumor cells were washed to remove NK cell, and counted by the trypan blue exclusion test.

To investigate whether the novel isolated anti-CTLA-4 mAbs affect the growth of tumor cells, SK-BR-3 cells (5 × 10^3^ cells/well), LNCaP cells (3 × 10^3^ cells/well) or MCF-7 cells were plated in 96-well flat-bottom plates for 16 h and then incubated, in the absence or in the presence of the indicated mAbs used at increasing concentrations, for 72 h. Cells were counted as described above and cell survival was expressed as percentage of viable cells in the presence of the drugs with respect to the untreated cells or treated with an unrelated IgG mAb, used as negative controls.

### 4.12. ADCC Assays and Determination of Cell Lysis

To clarify whether the novel isolated anti-CTLA-4 mAbs induce Treg killing by ADCC, NK cells (6 × 10^4^) were co-cultured with Treg cells or with control CD8^+^ or CD4^+^/CD25^−^ T cells (Effector ratio 3:1) in the absence or in the presence of ID-1, ID-8 or Ipilimumab mAbs, tested at the concentrations of 0.5 nM, 5 nM, and 20 nM. The ADCC induced by NK cells was evaluated by measuring the LDH released in the medium of co-cultures after 24 h of treatment, by using the LDH detection Kit (Thermofisher Scientific, Meridian Rd., Rockoford, IL, USA).

In order to test the cytotoxic activity of ID-1 and ID-8 mAbs, the LDH Cytotoxicity Assay was carried out also on co-cultures of CTLA-4-positive or CTLA-4-negative tumor cells with NK cells incubated as described above, by measuring the LDH released by tumor cells into supernatant after lysis, as previously reported [6,53].

### 4.13. Analyses of Cytokine secretion by Immune Cells

To investigate the capacity of the novel anti-CTLA-4 mAbs to induce cytokines secretion, ID-1, ID-8 or Ipilimumab mAbs were tested at the concentration range of 0.5–50 nM on unfractioned human or mouse lymphocytes, and human enriched NK cells stimulated with SEB or PHA (Sigma-Aldrich St. Louise, MO, USA), used at the concentration of 50 ng/mL and 2.5 μg/mL respectively, for 66 h at 37 °C. Untreated or stimulated cells were incubated in parallel in the absence or presence of an unrelated IgG, used as a negative control. The levels of IL-2, IFNγ or IL-6 in cell culture supernatants were measured by ELISA assays (all from DuoSet ELISA, R&D Systems, Minneapolis, MN, USA), according to the manufacturer’s recommendations as previously described [38,48].

To test the effects of the novel mAbs on co-cultures of tumor cells with NK cells, SK-BR-3 or MCF-7 tumor cells were co-cultured with NK cells and treated in the same conditions described above. Cell culture supernatants were analyzed by ELISA assays as mentioned above, to measure the concentration of IFNγ cytokine secreted in the medium.

### 4.14. Western Blotting Analysis of Cell Extracts

SK-BR-3 and LNCaP cells were plated at a density of 6 × 10^5^ cells/well in six-well plates and treated for 72 h with ID-1, ID-8 or Ipilimumab mAbs (400 nM). The enriched NK cells were plated at a density of 1 × 10^6^ cells/well in 48-well plates and treated with the above mentioned mAbs at the same concentrations for 66 h. After the treatments, cells were scraped and collected by centrifugation at 1200 rpm for 10 min. The cell pellets were resuspended in a lysis buffer of 10 mM Tris-HCl pH 7.4, 0.5% Nonidet-P-40, 150 mM NaCl, containing 1 mM Sodium orthovanadate (Sigma-Aldrich, St. Louise, MO, USA) and protease inhibitors (Roche, Indianapolis, IN, USA). After lysis, the protein concentration of cell extracts was determined by the Bradford colorimetric assay (Sigma-Aldrich, St. Louise, MO, USA) and Western blotting analyses were performed by incubating the nitrocellulose filters with the indicated commercial primary anti-phospho44/42 MAPK (indicated as pErk), anti-phospho-(Ser/Thr) Akt, anti-Cleaved Caspase-3 polyclonal antibody, anti-vinculin, anti-p-Tyr polyclonal antibodies or anti-actin antibody followed by HRP-conjugated secondary antibody used for the detection, as previously described [54].

### 4.15. Bioluminescent Cell-Based Assay

The bioluminescent CTLA-4 blockade cell-based bioassay (Promega, Madison, WI, USA) was performed by following the manufacturer’s recommendations. Briefly, antibody serial dilutions were prepared and added to the plated CTLA-4 effector cells (Jurkat T cells expressing human CTLA-4 and a luciferase reporter driven by a promoter responding to TCR/CD28 activation). The APC/Raji cells expressing an engineered cell surface protein designed to activate cognate TCRs in an antigen-independent manner and expressing CD80 and CD86 were added. The plates were then incubated for 6 h at 37 °C in a 5% CO_2_ incubator. The reagents provided by the kit were added as recommended at room temperature for 5–15 min, and the luminescence was measured by using a luminescence plate reader.

### 4.16. Statistical Analyses

Error bars were calculated by considering the results obtained by at least three determinations obtained in three independent experiments. Error bars depict means ± SD. Statistical analyses were assessed by Student’s t-test (two variables). Statistical significance was established as *** *p* ≤ 0.001; ** *p* <0.01; * *p* <0.05.

## 5. Conclusions

We report here for the first time on signaling pathways downstream CTLA-4 in tumor and NK cells revealed by using Ipilimumab, the approved FDA anti-CTLA-4 mAb, in parallel studies with two novel human mAbs we isolated by using an efficient phage display selection strategy for mouse-human CTLA-4 cross-reactivity. The novel antibodies, devoid of the antagonistic activity of Ipilimumab, but endowed with inhibitory effects on tumor growth and activatory effects on NK cells, could provide efficient anti-tumor effects through selective tumor and Treg cells killing by ADCC without the irAEs associated with the antagonistic activity of Ipilimumab.

## Figures and Tables

**Table 1:** Binding affinity of mAbs for CTLA-4 purified or expressed on lymphocytes. The Kd values obtained from the binding curves of the novel isolated anti-CTLA-4 mAbs on human or mouse CTLA-4-Fc recombinant protein and on activated human or mouse lymphocytes are reported in the table.

Kd Values				
mAbs	Human CTLA-4 Protein	Human Activated Lymphocytes	Mouse CTLA-4 Protein	Mouse Activated Lymphocytes
**Ipilimumab**	0.17 nM	0.32 nM	N. D.	N. D.
**ID1**	2.5 nM	0.22 nM	2.35 nM	0.45 nM
**ID8**	1.95 nM	0.34 nM	1.25 nM	0.62 nM

## Supplementary Materials

The following are available online at https://www.mdpi.com/2072-6694/12/8/2204/s1, Figure S1: Competitive ELISA assays of ligands and mAbs to CTLA-4; Figure S2: Competitive ELISA assay of ID1 or ID8 mAbs with biotinylated Ipilimumab; Figure S3: Effects of anti-CTLA-4 mAbs on isolated CD8^+^T cells and downstream pathways; Figure S4: Full length blots of Figure 7C,D; Figure S5: Full length blots of Figure 8A,B; Figure S6: Full length blots of Figure 9.
