# Supplementary material for: Isolation of Two Novel Human Anti-CTLA-4 mAbs with Intriguing Biological Properties on Tumor and NK Cells
Source: Cancers (Basel). 2020 Aug 6;12(8):2204. doi: 10.3390/cancers12082204 (PMC7464132; doi:10.3390/cancers12082204)
Supplement: Supplementary file 1 [file cancers-12-02204-s001.pdf]

Supplementary Materials

TITLE: ISOLATION OF TWO NOVEL HUMAN ANTI-CTLA4 mAbs WITH INTRIGUING BIOLOGICAL PROPERTIES ON TUMOR AND NK CELLS

Margherita Passariello[1,2], Cinzia Vetrei[1,2], Emanuele Sasso[2,3], Guendalina Froechlich[2,4], Chiara Gentile[1,2], Anna Morena D'Alise[3], Nicola Zambrano[1,2], Elisa Scarselli[3], Alfredo Nicosia[1], Claudia De Lorenzo[1,2].

Supplementary Figures

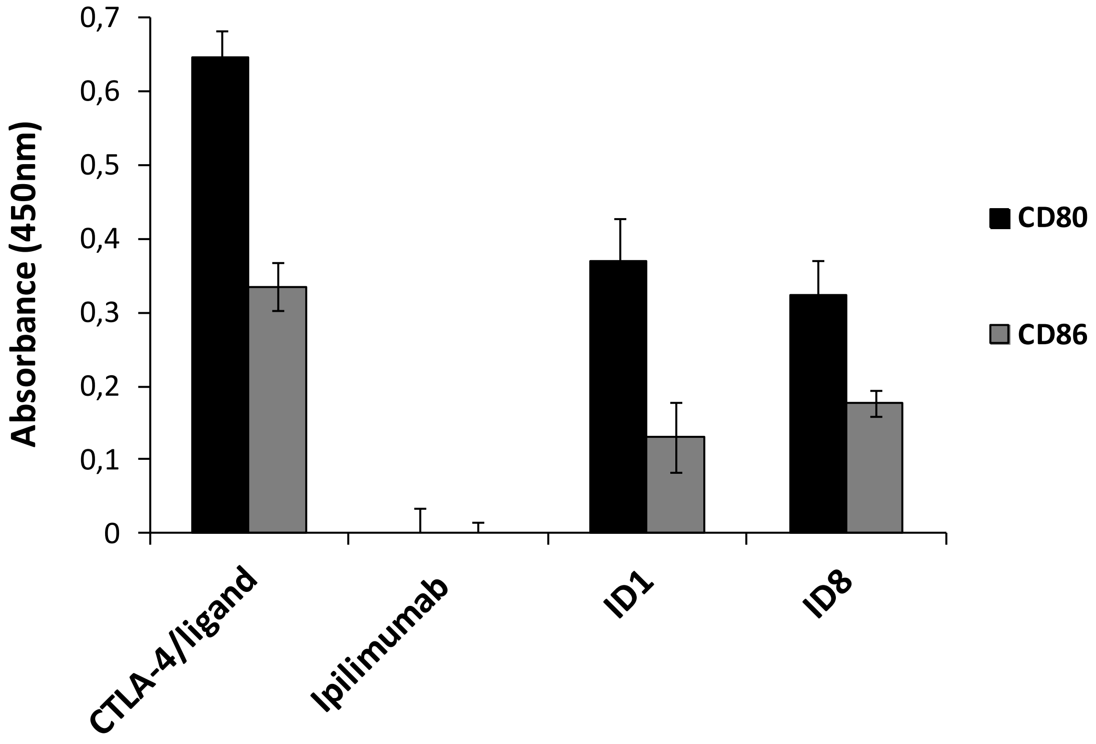

Figure S1 **Competitive ELISA assays of ligands and mAbs to CTLA-4.** Binding of CD80 and CD86 ligands to immobilized CTLA-4/Fc in the absence or in the presence of the indicated anti-CTLA-4 mAbs.

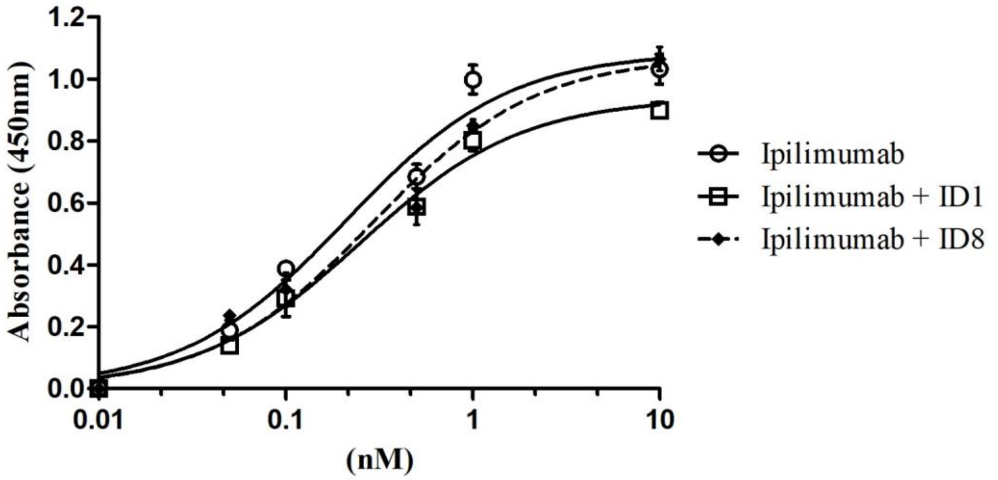

Figure S2. Competitive ELISA assay of ID1 or ID8 mAbs with biotinylated Ipilimumab. Competitive ELISA assays were performed by measuring the binding of biotinylated Ipilimumab to CTLA-4 recombinant protein at the indicated concentrations in the absence (empty circles) or in the presence of the unlabeled ID1 (empty squares) or ID8 (full circles) mAbs used at saturating concentration (400 nM), respectively. Binding values were reported as the mean of at least three determinations obtained in three independent experiments. Error bars depicted means ± SD.

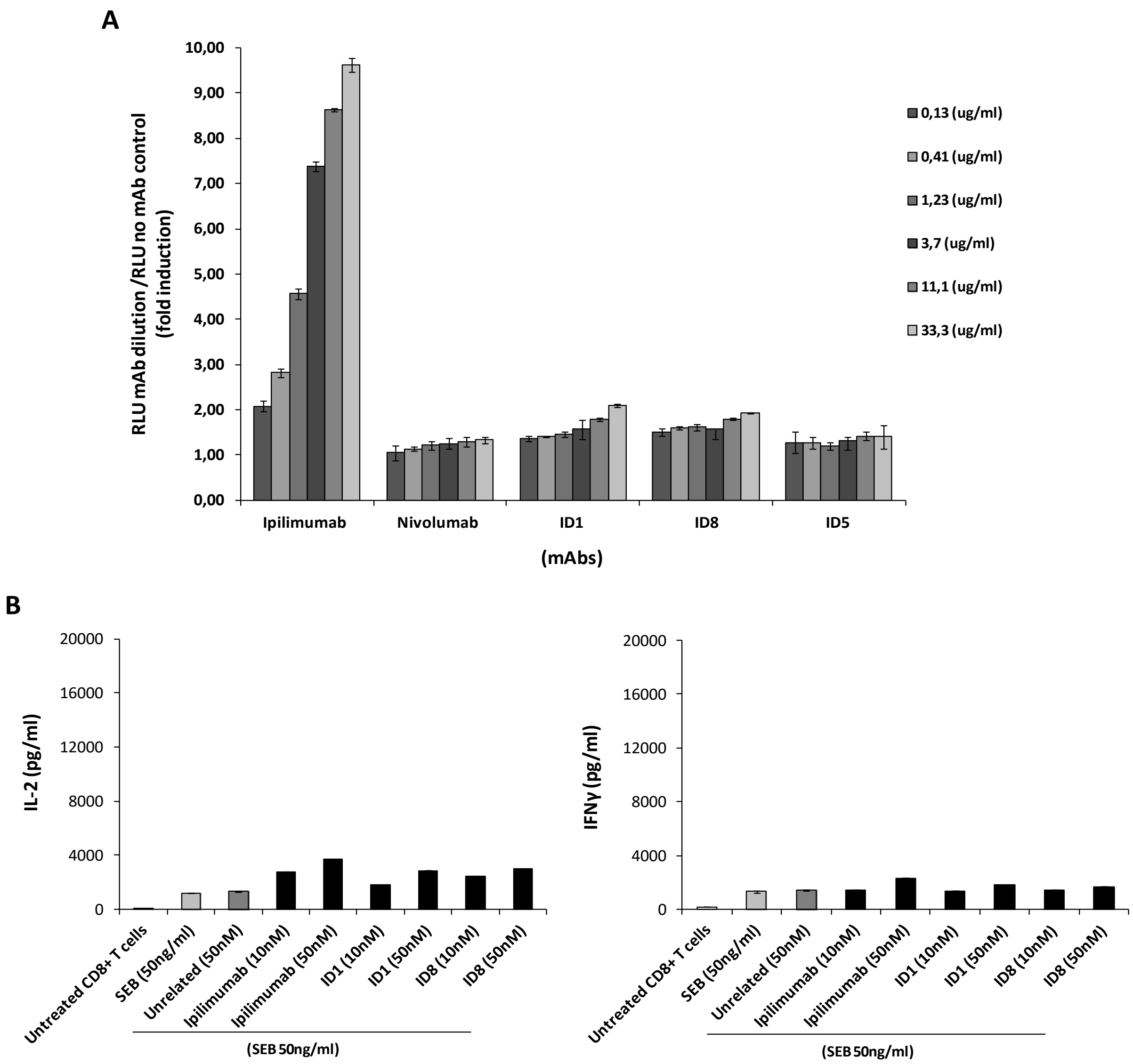

Figure S3. Effects of anti-CTLA-4 mAbs on isolated CD8^{+}T cells and downstream pathways. (A) Bioluminescent CTLA-4 blockade cell-based bioassay. Effector Jurkat T cells were co-cultured with APC Raji cells and incubated in the absence or in the presence of the indicated antibody serial dilutions, for 6 hours at 37°C. Luminescence values were reported as the mean of at least three determinations obtained in three independent experiments. **(B) Effects of the mAbs on cytokine secretion by isolated CD8^{+} T cells.** CD8^{+} T cells, isolated as described in the Methods, were incubated with Ipilimumab, ID-1 or ID-8 mAb at the indicated concentrations in the presence of SEB (50 ng/mL) for 66 hours at 37°C. The levels of cytokine secretion were evaluated by ELISA assays on supernatants of the treated cells. Ipilimumab and an unrelated IgG antibody were used as positive or negative control, respectively. Error bars depicted means ± SD.

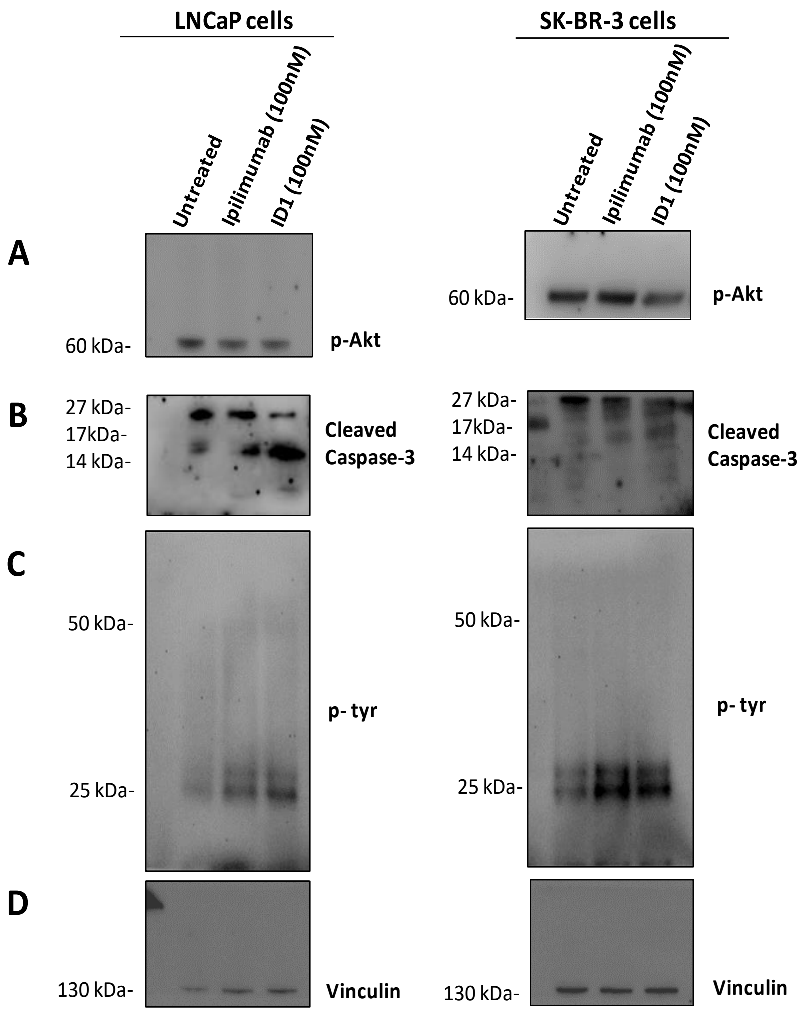

Figures S4 Full length blots of Figure 7 C and D. The different portions of the same filter were stained with: (**A**) the anti-phospho-(Ser/Thr) Akt polyclonal antibody (Cell Signaling Technology, Danvers, MA, USA); (**B**) the anti-Cleaved Caspase-3 polyclonal antibody (Cell Signaling Technology, Danvers, MA, USA); (**C**) the anti-p-Tyr monoclonal antibody (Santa Cruz Biotechnology, Inc. Dallas, Texas USA); (**D**) the anti-vinculin monoclonal antibody (Santa Cruz Biotechnology, Inc. Dallas, Texas USA). The intensity of the bands was normalized to vinculin.

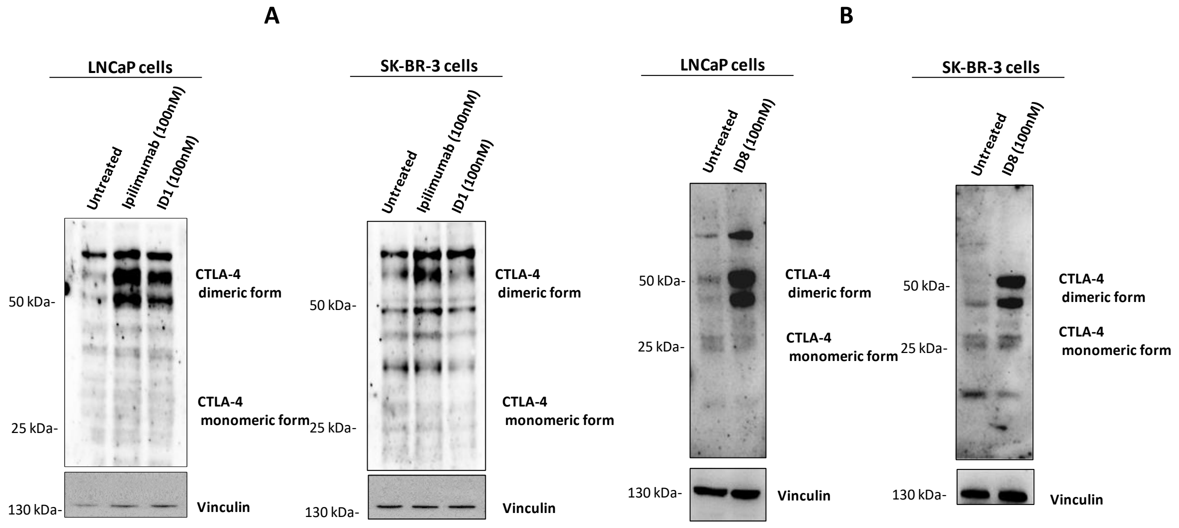

Figure S5 Full length blots of Figure 8 A and B. The filter was stained with: the commercial human anti-CTLA-4 polyclonal antibody (R & D Systems, Minneapolis, MN, USA) and with the anti-vinculin monoclonal antibody (Santa Cruz Biotechnology, Inc. Dallas, Texas USA). The intensity of the bands was normalized to vinculin. The ratio of dimeric/monomeric form of CTLA-4 was calculated after normalization.

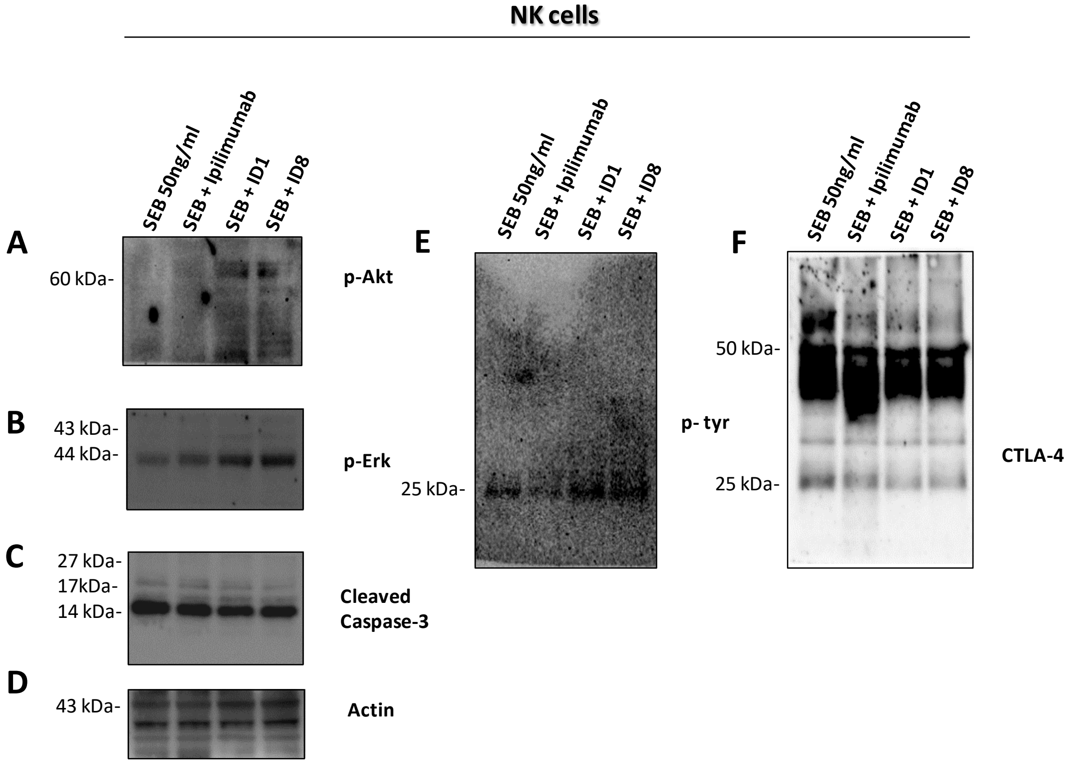

Figure S6 Full length blots of Figure 9. The filter was stained with: (**A**) the anti-phospho-(Ser/Thr) Akt polyclonal antibody (Cell Signaling Technology, Danvers, MA, USA); (**B**) the anti-phospho-p44/42 MAPK (Erk1/2) polyclonal antibody (Cell Signaling Technology, Danvers, MA, USA); (**C**) the anti-Cleaved Caspase-3 polyclonal antibody (Cell Signaling Technology, Danvers, MA, USA); (**D**) the anti-actin monoclonal antibody (Sigma-Aldrich, Darmdstadt, Germany); (**E**) the anti-p-Tyr monoclonal antibody (Santa Cruz Biotechnology, Inc. Dallas, Texas USA) and with (**F**) the commercial human anti-CTLA-4 polyclonal antibody (R & D Systems, Minneapolis, MN, USA). The intensity of the bands was normalized to vinculin.
